# Evaluation of a bridge-based suicide intervention programme: findings from a qualitative study with volunteers and professional staff

**DOI:** 10.1186/s12889-026-26849-9

**Published:** 2026-03-11

**Authors:** Nicholas Woodrow, Eleanor Holding, Olivia KL Hamilton, Peter Craig, Joe Hulin, Claire Giraud, Andrew Trathen, Paul Maloney, Leandro Garcia

**Affiliations:** 1 Sheffield Centre for Health and Related Research (SCHARR), University of Sheffield, Sheffield, S1 4DA UK; 2 Health Economics and Health Technology Assessment, School of Health and Wellbeing, Glasgow University, Glasgow, G12 8TB UK; 3 City and Hackney Public Health Team, London Borough of Hackney, Hackney, E8 1DY UK; 4 Ascension Trust, Alpha House, Alpha Place, Surrey, SM4 4TQ UK; 5 Centre for Public Health, Queen’s University Belfast, Belfast, BT12 6BA UK

**Keywords:** Suicide prevention, Suicide intervention, Volunteer programmes, Bridges, Public spaces, Qualitative research, Public health

## Abstract

**Background:**

Suicide is a preventable, but complex public health issue. Around one-third of suicides take place in a public location. Easy access to means of self-harm in public spaces, such as bridges, increases risk. While physical modifications (e.g., barriers) are effective for means reduction, they are not always feasible to implement. Bridge Watch is a suicide prevention initiative that uses trained volunteers to patrol public spaces around bridges in the City of London. The volunteers aim to identify and engage with individuals in distress to reduce suicide incidents (attempts and suicide deaths).

**Methods:**

This qualitative study explores the implementation barriers and facilitators, and the perceived effectiveness of Bridge Watch. We conducted 27 semi-structured interviews with professionals who have had some involvement with Bridge Watch (*n* = 8) and current Bridge Watch volunteers (*n* = 19). Data collection was supplemented by analysing 16 volunteer diary entries completed post-patrol. Data were analysed using framework analysis.

**Results:**

Volunteers and professionals reported examples of how the Bridge Watch programme had identified and supported individuals in acute crises, as well as intervening with people who might otherwise have gone unnoticed. The programme was seen to provide a range of secondary public health impacts, including supporting and signposting for vulnerable individuals, and raising public awareness of suicide prevention. Emergency service staff suggested that Bridge Watch can help reduce pressure on services by de-escalating situations. Robust, relevant and frequent training which reflects the conditions of the Bridge Watch role is crucial for preparing volunteers. The Bridge Watch programme faces several operational challenges, including volunteer recruitment and capacity to provide 24-hour coverage. While positive relationships exist with statutory services (e.g., police), there is a need for improved awareness and stronger integration.

**Conclusions:**

Bridge Watch appears to be a promising, acceptable, and valued suicide prevention strategy. It offers benefits through direct interventions and broader public health contributions. Its key strengths are its proactive, visible, immediate, human-centred approach and its ability to support statutory services. However, in the development of such interventions, consideration needs to be given to potential unintended consequences, including the risk of drawing attention to specific locations as sites of suicide.

**Supplementary Information:**

The online version contains supplementary material available at 10.1186/s12889-026-26849-9.

## Background

Suicide is a preventable, but complex public health issue which results in a substantial number of deaths, both globally [1, 2] and within England and Wales [3, 4] each year. In 2023, approximately 6000 suicides were recorded across England and Wales [4]. Suicide has devastating consequences affecting families, friends, witnesses, first responders, and wider communities [5–7], and has significant resource and financial costs for services [3].

Across England and Wales, around one third of suicides take place in a public location [8, 9]. Therefore, efforts to reduce and prevent suicide in public spaces have emerged as crucial aspects of suicide prevention strategies [10], with a focus on local authorities to identify risk sites and to take action to prevent suicides at these sites [9]. Whilst many factors contribute to suicidal ideation and public suicide attempts, easy access to methods of self-harm, like bridges to jump from, can significantly increase the risk of suicide incidents [11].

Even though London had the lowest rate of suicides registered of any region in England in 2023 (8.3 deaths per 100,000) [4], historical data from the City of London suggests that between 2009 and 2014, the most frequently used methods of suicide were drowning in the River Thames (32%) and falling from a height (26%) [12]. This pattern contrasts with national data, which identifies hanging, strangulation, or suffocation as the predominant method across England and Wales [13]. Although more recent or borough-level data disaggregating suicide methods within London are not publicly available, these data underscore the need for geographically targeted suicide prevention strategies, particularly those focused on bridge and river-related environments. Indeed, bridges are seen as specific priority sites for suicide prevention due to their accessibility [11, 14]. Further, the public nature of bridge-based suicides and the media coverage of suicide events can lead to contagion effects, further exacerbating issues [11, 15–18]. This makes such sites a significant concern [3], and implementing effective interventions here, crucial.

Typically, approaches to bridge-based suicide interventions include a mix of physical modifications such as nets, fences and barriers to restrict access to means [14, 19], placing emergency telephones or crisis line signage on/around bridges to encourage help-seeking [11], and increasing the likelihood of a person being identified and stopped through CCTV/camera surveillance [3, 11]. Reducing access to means of self-harm through the implementation of physical barriers or nets on bridges has consistently demonstrated effectiveness in reducing suicides [11, 14, 19]. Importantly, research suggests that physical barriers do not simply displace suicides to other locations or methods [6, 15, 18, 20]. While effective, the installation of physical barriers for suicide prevention can be costly and controversial. They often face opposition due to aesthetic concerns, negative media portrayals, and the challenges of securing permission to build on historic sites and buildings [14, 18]. Thus, such approaches are not always feasible.

Whilst there is good evidence of the effectiveness of physical modifications on bridges, there is less evidence around the effectiveness of other approaches [11], such as human intervention on bridges [21]. A small number of studies have looked at ‘bystander’ or ‘third-party’ interventions in public spaces (e.g., at railways [22]), and show the potential and value of human intervention in preventing suicide. Studies reveal that interventions by members of the public can effectively ‘burst the bubble’ of acute suicidal ideation [21]. However, spontaneous intervention can be low due to public concerns about causing harm and being unsure how to act [22, 23].

Considering the difficulty of implementing means restriction on all bridges (e.g., prohibitive costs, aesthetic concerns, and preservation requirements for historic sites), and the value of human interaction in interventions but reticence from members of the public to intervene in public spaces, alternative approaches to suicide prevention and intervention are important to explore. One such approach is to train staff or volunteers to actively patrol specific public spaces, to proactively identify and compassionately engage with individuals in need. The Bridge Watch programme is an example of this model. Below, we describe Bridge Watch programme drawing on the Template for Intervention Description and Replication (TIDieR) checklist and guide [24].

### Bridge Watch

Bridge Watch is a suicide prevention initiative co-founded by the City of London Corporation’s (CoL) Suicide prevention lead and the Royal National Lifeboat Institution (RNLI) regional water safety lead and part of an action in the 2016-19 City of London Corporation Multi-Agency Suicide Prevention action plan, in response to the number of suicides and suicide attempts occurring on the City of London bridges, and the strain these incidents place on service resources. It was developed in collaboration with various stakeholders, including the Tidal Thames Water Safety Forum, Thrive LDN, the City of London Police, and the Ascension Trust. Bridge Watch was initially funded by City Bridge Foundation, and is delivered by Ascension Trust, collaborating closely with partners including the CoL, RNLI and emergency services.

The Bridge Watch programme deploys volunteer teams to patrol five bridges in the City of London, and aims to provide a physical presence of suicide prevention and intervention. The five bridges are major public bridges in the City of London. These bridges are mixed-use environments, characterised by both heavy vehicular traffic alongside high-footfall pedestrian walkways. The bridges are covered by CCTV surveillance. While police officers and park guards maintain a presence their patrols are not systematically related to suicide risk or specifically tasked with proactive suicide prevention.

Volunteers are provided with a comprehensive training package which supports them in delivering the role. Each volunteer is initially required to complete an online course accredited by the Zero- Suicide Alliance. They are then provided with a half-day training session delivered by the Listening Place and accredited by the Thames Skills Academy. This training covers an understanding of suicide and crisis, and, information on how to deliver an intervention (observation, key questions to ask, active listening, support options) with role play/simulation exercises. Volunteers also have access to additional training options (e.g., a two-day ASSIST training course).

The primary objective of Bridge Watch is to reduce suicide incidents (attempts, contemplations and suicide deaths) on the City of London’s bridges. This is done through an early intervention approach, engaging with individuals showing signs of intent to enter the water. During the patrols, the volunteers aim to identify people at risk of entering the water and then engage in empathetic listening, providing support, signposting and connection to appropriate resources, including emergency services intervention if needed. Patrols typically involve volunteers who wear Bridge Watch-branded coats, working in teams of two or three, with different teams having set routes to follow across the five bridges. Patrols involve a shift leader who organises and manages patrols. Each patrol lasts around two hours and is arranged around volunteer availability. Volunteers complete incident reports and shift logs after each patrol. Volunteers are expected to commit to a minimum of four patrol hours per month. The Bridge Watch programme employs one paid staff member, the Bridge Watch programme lead.

The first patrols began as a pilot in December 2023, with an increase in the number of patrols and expansion to patrolling five bridges occurring from April 2024.

### Research aims

This paper has been developed from a wider mixed-methods evaluation exploring the feasibility, acceptability and effectiveness of the Bridge Watch Programme. This paper draws specifically on the qualitative arm of the evaluation, which explored whether Bridge Watch is perceived as acceptable and effective from the perspectives of volunteers delivering the programme and professional stakeholders with varying degrees of involvement, including external agencies such as local emergency services that engage with the initiative. A complementary paper presenting quantitative findings on the impact of Bridge Watch on suicide-related incidents is currently in development.

Our qualitative study aimed to explore the implementation barriers and facilitators, and the perceived effectiveness and impact of the Bridge Watch programme. To our knowledge, this is the first evaluation of a suicide prevention initiative that employs an in-person volunteer presence, representing a novel contribution to the evidence base on suicide prevention. Given the limited research examining the effectiveness and impact of volunteer-led suicide prevention initiatives in public spaces [21], a qualitative approach was adopted to gain an in-depth understanding of stakeholder perspectives and implementation processes. This study contributes to the ongoing development of evidence-informed strategies to prevent suicides from bridges and in public spaces.

## Methods

We conducted semi-structured interviews with professionals and volunteers, alongside collecting volunteer diary entries completed after patrols.

Between August and September 2024, we undertook 27 interviews with:

- i) Volunteers who undertake Bridge Watch patrols (n=19)
- ii) Professionals who have had some involvement with Bridge Watch (n=8), including Bridge Watch staff, emergency service workers (police officers), street triage mental health nurses (who accompany police officers to incidents where people need immediate mental health support), park guards (a London-based service that collaborates closely with local authorities and police to deliver community safety services), and Public Health Practitioners.

While interviews were conducted in August and September, the sample included professionals and volunteers who had been involved since Bridge Watch’s inception in November. Consequently, the data captures retrospective accounts covering different seasonal periods.

Between August and November 2024, we also collected 16 diary entries from four Bridge Watch volunteers.

### Participant recruitment

We used purposive and convenience sampling to recruit study participants. Recruitment and dissemination of study information to volunteers and professionals were facilitated by our project partners in the Bridge Watch team. Professional staff were provided with a verbal description of the research, an information sheet and consent forms (see Supplementary file) and invited to contact the research team to participate.

Volunteers were approached by the Bridge Watch coordinator, who provided a verbal description of the study. If volunteers expressed interest in participation, a full project information sheet was provided. All volunteers were provided with time to read the information sheet and to ask questions about the study and their participation. All volunteers consented to their contact information being securely transferred to the research team. The research team contacted potential participants to further discuss the research and then followed up after a week to arrange interview times. At the end of the interviews with volunteers, each was invited to take part in the diary entry aspect of the research. Those who expressed interest were sent a separate information sheet and a consent form (see Supplementary file), before participation.

All participants provided written informed consent for their involvement in the research, which was electronically signed.

### Data generation

All interviews took place via an online video call or telephone call. Interviews were undertaken by NW and EH and lasted between 40 and 80 min.

The interviews employed semi-structured topic guides (see Supplementary file). Interviews with professionals explored perceived effectiveness and outcomes of the Bridge Watch, and suggestions for improvement. Interviews with volunteers explored their perspectives on the training provided, discussed patrols and interactions on the bridges, perspectives on the effectiveness of the programme, and what could be improved.

Information relating to data management, confidentiality, right to withdraw and consent were verbally reaffirmed before data collection began. All interviews were audio-recorded only, using an encrypted recorder, and transcribed verbatim. All transcripts were anonymised at the point of transcription, checked for accuracy by NW and EH, and securely stored.

The volunteer diary logs involved volunteers completing a brief template following a series of four patrols. A template for the diary logs was developed to cover general reflections on patrol sessions (see Supplementary file). Completed diary logs were sent to the research team as password-protected Word documents. These were checked and anonymised by NW and EH, and securely stored.

We worked with a PPIE (Patient and Public Involvement and Engagement) group of six Bridge Watch volunteers to help shape and refine our data collection tools, and to sense check our findings during analysis to ensure they accurately reflected their experiences.

### Data analysis

Data analysis was informed by framework analysis [25, 26]. Framework analysis was selected as it is widely used in applied health research where research questions are defined in advance, multiple stakeholder perspectives are involved, and findings are intended to inform service development and policy [26]. NW and EH familiarised themselves with the data by independently reading over a selection of transcripts and diary logs, making notes on key points and ideas to identify initial codes. NW and EH then met to discuss and developed an initial coding framework, which was applied and revised using a selection of further transcripts and diary logs. The coding framework was revised following discussions between NW and EH, with the codes examined, merged, and grouped into potential themes and sub-themes. The transcripts and diary logs were then split between NW and EH, and the framework was imported into NVivo-14 and applied to all transcripts and diary logs. Following coding, the framework matrices were exported into Excel for charting. Here, NW and EH summarised and synthesised the data within each category, with the participants’ accounts summarised narratively alongside quotes. Finally, these summaries were then brought together and combined into overarching themes, with key quotes being used to support key points.

This was then used as a starting point for theme development of this paper, to address the paper’s aims of exploring the implementation barriers and facilitators and the impact and perceived effectiveness of the Bridge Watch programme.

### Ethics

Ethical approval for the study was granted by the Sheffield Centre for Health and Related Research (SCHARR) ethics committee at the University of Sheffield (Reference Number 062661).

## Findings

Data analysis generated three key overlapping themes, which provide insight into the perceived effectiveness, and implementation barriers and facilitators of the Bridge Watch programme: (i) Volunteer knowledge, training and participation in interventions shapes preparedness and confidence to intervene; (ii) Operational challenges impact the reach and delivery of the programme; (iii) Achieving perceived impact and benefits beyond suicide prevention.

We use verbatim quotes from participants to illustrate the key findings. For each quote, we detail the type of participant (e.g., professional practitioner with a general job role, and volunteer with participation number).

### Theme 1: Volunteer knowledge, training and participation in interventions shapes preparedness and confidence to intervene

The importance of robust training for volunteers was noted as crucial in developing awareness and preparedness for the role of suicide intervention around the City of London bridges. Whilst volunteers reported that the initial training they received (which was delivered in a classroom setting, and based on 1-2-1 active listening discussions) was beneficial and insightful, it also lacked specificity for the challenging, specific and dynamic conditions during bridge patrols and interactions (i.e., these can be busy and noisy environments, where making eye contact and listening can be challenging), with the training seen to be more preventative than intervention-focused (i.e., designed for engaging individuals actively asking for support and seeking connection, rather than managing acute crises where an individual is actively attempting to enter the water):

‘[in a quiet room] you can have a detailed conversation with someone, that’s heard. Whereas, when you intervene on a bridge, you’re in central London, so it’s noisy, there’s traffic going by, there’s members of the public. The person you’re intervening with is often, either distressed, they can be shouting or there’s some sort of substances involved…They can walk away, which doesn’t happen in a classroom, or run away. And there is actual physical danger present. So, the person could jump over while you’re talking to them. So, that is really different than being trained in a classroom where there’s no physical danger or jeopardy.’ (Volunteer 15, interview).

This quote highlights how the physical environment of the bridges acts as an important variable in interventions. Unlike clinical settings or private locations, the bridges are complex and high-stimulus environments, with traffic, pedestrians, adverse weather and noise. This creates barriers in identifying issues. The in-situ role play/simulation training that was provided was seen as extremely beneficial to support preparedness of the role:

‘What has been much more useful to me is the roleplaying that we’ve done on the bridges…[we] did some roleplaying which felt really realistic and was actually sort of terrifying. And that was much more useful…You have to be realistic about what it’s actually like on the bridges, and so the roleplay is much more useful.’ (Volunteer 12, interview).

Further, a large amount of learning was described as being gained from undertaking patrols with more experienced volunteers, and using continued role play/simulation:

‘On my first patrol, I was out with [volunteer] and [volunteer]…I went out with them, and they were amazing…we also did some roleplays, like, out on the bridges. So, we did a roleplay where [name] was kind of, in the role of someone who is kind of, struggling on the bridge, and I did, like, an intervention, and then we’d get [name] in to help. So, that was really good to kind of, practice.’ (Volunteer 7, interview).

There was a general positive perception of the overall training, with reflections that it was providing volunteers with a foundation of knowledge for delivering interventions. However, during patrols, there was only a small number of actual suicide interventions. Indeed, across the 16 patrol diaries we collected, only one intervention-related incident was recorded (this is an illustrative example rather than a measure of intervention frequency, as we did not have the data to permit quantification of intervention frequency). For some volunteers, the infrequent nature of interventions was reported as having a detrimental impact on their skills and confidence to intervene:

‘When there’s long gaps between intervention work, you can get rusty quite quickly because you’re not doing enough of it.’ (Volunteer 1, interview).

Due to the ‘high-stakes’ nature of interventions and a fear of error from volunteers, intervention roles often naturally formed, with leadership during interventions predominantly falling to more experienced volunteers. While this dynamic leveraged existing expertise, it inadvertently limited practical experience opportunities for less experienced volunteers, potentially perpetuating a cycle that hindered skill and confidence development:

‘My role is not going to be talking to somebody because I’m usually with someone who’s more experienced…We’re not mucking about, it’s not a training exercise, it’s not a chance for me to get a bit of experience, so I tend to hang back.’ (Volunteer 10, interview).

Volunteers reflected on the experienced and potential personal and emotional impact of participating in patrols and in interventions. They highlighted the importance of ongoing formal and informal support (from other volunteers), and supervision from the Bridge Watch coordinator due to the potentially distressing nature of their roles.

‘The importance really of gathering together at the beginning, gathering together at the end, so that you can make sure everybody goes home okay, anything anybody wants to talk about. Because we have to do some hard things sometimes, and it’s good to talk that though and check.’ (Volunteer 10, interview).

‘It was helpful to talk it [the intervention] through after with others on the shift to debrief.’ (Volunteer 2, diary entry).

### Theme 2: Operational challenges impact the reach and delivery of the programme

Various operational challenges impacted the Bridge Watch programme. Providing patrol coverage over the large geographic area of the bridges was a key issue as volunteers cannot physically be present at every bridge simultaneously. While volunteers are granted a degree of agency to determine their specific patrol routes based on real-time assessments, they must cover key areas during the patrol:

‘I think the biggest challenge is the distance between the bridges, the lost time of it. And we’ve dabbled between hanging around bridges for longer, and then moving onto the next, to trying to get as many laps in as possible, and I don’t know which one is the right approach.’ (Volunteer 8, interview).

Further, trying to provide 24-hour cover from Bridge Watch was noted as a significant challenge, especially with current volunteer levels, and the need to find volunteers to cover unsociable hours (i.e., hours through the night and early morning).

During patrols, the difficulty of identifying individuals’ ‘at risk’ within what can be extremely busy environments was a challenge:

‘It’s just spotting those that need the support is massively difficult in the environment that we are and in the location that we are, its tourist hotspots, lots of people taking photos, lots of people standing out, looking and on their phones, handsfree now. So, I think just the location of where we are makes it really difficult to spot people in crisis, unless they are standing there in tears or sitting on the edge, it’s really very difficult.’ (Volunteer 7, interview).

Weather conditions and the physical demands of the role were discussed by volunteers. While the distance covered during patrols provided the benefit of physical activity for some, it also presented a challenge for others:

‘It’s physically demanding…and I think that is a challenge sometimes…you know, you’re not kind of just sitting around on a bridge on a chair waiting for something to happen, you’re on your feet a lot.’ (Volunteer 1, diary entry).

In relation to wider awareness of Bridge Watch, the volunteers noted the programme was developing good links with other services that work on and around the bridges (e.g., police officers, park guards). This was also reported by park guards, who spoke of the benefit of informal information sharing during crossovers on patrols. Interviewees from the police and emergency service workers who had engaged with Bridge Watch were extremely positive about the programme and the support and role it can provide. However, several professionals suggested that the police and other services lacked awareness of Bridge Watch:

‘I don’t know if any of my colleagues have any dealings other than me. I didn’t know they were a thing until I spoke to them on the first time I met them, and they, sort of, explained what they did. And I was like oh…because we never really hear about it.’ (Professional practitioner - Police Officer).

‘I’ve been aware of it from the very beginning…I would say some of the response officers are not aware of them, because they don’t have any interaction with them.’ (Professional practitioner - Street Triage Mental Health Nurse).

Due to Bridge Watch being a relatively new and small service, it was suggested that further engagement work was required to better embed Bridge Watch within the wider network of support. However, the time capacity of the Bridge Watch coordinator was identified as a key challenge which prevented promotion and expansion of the programme.

### Theme 3: Achieving perceived impact and benefits beyond suicide prevention

All volunteers and professional staff perceived the Bridge Watch programme as having a positive impact in terms of preventing suicides. It was acknowledged by both volunteers and professional staff that it is extremely difficult to demonstrate the impact of Bridge Watch, due to the challenge of detecting if someone would go on to enter the water without intervention. Nevertheless, there was a perception from the volunteers that their work made a difference, with examples of where Bridge Watch had encountered and supported people, potentially preventing them from entering the water:

‘We definitely have helped people, stopped them from entering the water, signposted care that they wouldn’t have known about.’ (Volunteer 15, diary entry).

‘I think it works quite well…[the] times they’ve called us we’ve actually needed to be there…if we weren’t called, that person probably would have gone in the water.’ (Professional practitioner - Police Officer).

A key strength of the Bridge Watch programme was seen to be the proactive nature of the initiative and the physical presence of volunteers to provide direct support via human intervention. Further, the Bridge Watch volunteers spoke of intervening with people who did not express any overt signs of distress, but who were identified from more subtle observation and instinct. This highlights the volunteers’ potential to detect and intervene with individuals who might otherwise have gone unnoticed, demonstrating the unique value of the Bridge Watch programme in preventing potential suicides:

‘You get that feeling when you know that somebody’s not right, not happy…we saw someone just standing around, they didn’t look in distress, but you just think, there’s something not right here…and they were one of the ones who needed the services calling, as they were a risk of going in the water. And it wasn’t obvious until we started talking to them.’ (Volunteer 13, interview).

A large amount of the volunteers’ role was described as delivering wider public health support beyond suicide prevention. This included: risk management of people doing ‘unsafe’ things on/around the bridges (e.g., sitting on the bridge ledges); supporting vulnerable, intoxicated and distressed people; and assisting partner agencies and other services in managing incidents (e.g., the police in looking for missing people). Thus, the benefits were seen to go beyond suicide prevention:

‘It’s having lots of public health impacts, stopping people doing unsafe things like sitting on the edge of the bridges for photos, and being able to signpost people who are struggling or have an issue, to services to help them. But that’s all really hard to measure.’ (Professional practitioner - Public Health Practitioner).

’We don’t just focus entirely, although it’s our main function, on people who are looking suicidal. But you see, you just see people in some sort of trouble, argument, or something, and you will engage, you know. You will engage beyond your specific brief.’ (Volunteer 7, interview).

The patrols were discussed as acting as a form of suicide prevention through the visibility of volunteers. Indeed, many volunteers spoke of the importance of the role as a physical presence in the de-stigmatising of suicide, alongside raising awareness of issues and support:

‘So, just being there, having the jacket on, you know, that says, Bridge Watch, and trying to hopefully help people feel a bit safer, even if they don’t interact with us…It’s actually doing interventions, and then there’s also that kind of, preventative part of just being someone who could be there, if someone, you know, was having a bad day. And also, sharing, kind of, a message around, like, that suicide is important…and letting them know that people do care in London, because it can be quite an unfriendly place.’ (Volunteer 6, interview).

Volunteers spoke of the positive reaction from the public to Bridge Watch and their presence on the bridges. Bridge Watch was seen as being perceived differently to emergency services by members of the public. This distinction was noted to be particularly beneficial in helping build rapport, stabilise situations and engage people who may have been reluctant to engage with emergency services:

‘It just helped that it was another friendly face that wasn’t a uniformed presence because sometimes with us being a uniform presence, people can go one of two ways. They can either go, you are a uniform presence, please help me or it can go, you are a uniform presence, I don’t like you, so I don’t want to talk to you. So, it was nice just to have someone through the Bridge Watch service, just wearing a plain green coat and a baseball cap saying, talk to me, tell me what’s going on.’ (Professional practitioner - Park Guard).

Professional staff perceived Bridge Watch as contributing to reduced pressure on the police/emergency services around the bridges, by preventing callouts and de-escalating issues before emergency service involvement:

‘So, they, sort of, ease the pressure on the police. Basically, like a mini, like a pre-triage, I suppose.’ (Professional practitioner - Street Triage Mental Health Nurse).

‘They’ve got people away from the bridge in some cases, which is a massive thing. Because if someone’s on the bridge, all the resources…we get the marine units out, ambulance, everyone. Everyone’s out in case that person jumps.’ (Professional practitioner - Police Officer).

A wider concern from some professional staff was that Bridge Watch would increase the use of emergency services, as volunteers would call the police to help manage their anxiety around any ‘risky situations’. However, this was not suggested to have been observed among the interviewed emergency service professionals:

‘I think my initial reaction to finding out about it [Bridge Watch] was like, oh, great, so we’re just going to get more calls to the bridges, because they’re going to find the people. But actually, I think there’s been a reduction…it definitely feels like there’s been less calls for services to the bridge…I think it works quite well in that respect, that they’ll only call us if there’s an actual need for a police officer to attend, like if that person’s life will end otherwise. And if not, I think they’re quite good at leaving us out of it and just cracking on.’ (Professional practitioner - Police Officer).

## Discussion

Our findings suggest that Bridge Watch is seen to provide a proactive, visible presence on the City of London bridges, which is helping to identify and support individuals in acute crises. Volunteers and professionals reported examples of how the Bridge Watch programme had supported and prevented potential suicides and harm for people entering the water, as well as intervened with people who might otherwise have gone unnoticed. Robust, relevant and frequent training which reflects the conditions of the Bridge Watch role is crucial for preparing volunteers. While training can provide a good knowledge base, the infrequency of real-world interventions, coupled with the high-stakes nature and fear of error, can lead to less experienced volunteers having limited opportunities to practice their skills and build confidence, with intervention leadership often defaulting to more experienced volunteers. The Bridge Watch programme faces several operational challenges, including volunteer capacity to provide 24-hour coverage across the City of London bridges, and identifying individuals at risk in busy environments. The programme has established positive relationships with other services like the police, but there’s a recognised need to improve awareness and better integrate Bridge Watch within wider support networks.

### The role and added value of Bridge Watch in suicide prevention

The Bridge Watch programme’s use of trained volunteers to patrol suicide risk areas to directly intervene with people, presents an alternative way of approaching and delivering suicide intervention in public spaces. Thus, it differs from typical and more conventional suicide prevention initiatives (e.g., ‘hotline’ support such as the Samaritans [27]), which require individuals actively seeking help and support, as it proactively attempts to engage people in immediate need, even if they have not actively or directly requested or sought support. Further, it goes beyond restricting access to means and introducing physical modifications (e.g., bridge barriers/nets) as it creates a human presence at at-risk sites. The volunteers’ presence and ability to identify and distinguish subtle cues of distress within the complex environments of the bridges is a key element of Bridge Watch’s value. Bridge Watch’s support and signposting approach, alongside its direct link to services, provides an active pathway into support for those who are in need. It does not rely on deterrence as a mechanism for the prevention of suicides but actively seeks to identify and provide support. Overall, our study suggests that volunteer presence on the bridges may support suicide prevention [28].

In addition to direct intervention around suicide and preventing people from entering the water, Bridge Watch is seen to be providing a range of secondary benefits and positive public health impacts, including risk management around the bridges, signposting for vulnerable individuals, raising public awareness of suicide and suicide prevention, and assisting partner agencies like the police in managing incidents. By de-escalating situations and providing initial support, Bridge Watch volunteers were seen to prevent unnecessary callouts and reduce the workload on police and other emergency services. The volunteers’ abilities to establish rapport with individuals was crucial. The importance of displaying warmth and genuine concern has been noted as significant in crisis intervention [29], and was seen as a key in our study, as was a separation and distinction from services such as the police. Thus, our findings suggest that volunteer-led interventions in public spaces, if positioned and use appropriately, may complement existing support and can reduce use of emergency services.

Given the multifaceted nature of suicide, effective prevention requires coordinated, multi-level approaches that integrate efforts across different sectors [1, 10, 30, 31]. In relation to bridge-based suicide intervention approaches, the challenge of identifying need, encouraging public support in public spaces [22, 23], and the challenge of being unable to implement physical barriers [14, 18, 19], means there is a need for alternative approaches. Whilst surveillance technologies and AI-powered systems have been suggested as aiding the detection of potential suicide attempts on bridges [3], there is still a need for a timely and effective human response. Here, a programme like Bridge Watch appears to have great potential to contribute to a more holistic approach. Further, our findings highlighted a key strength of volunteer-based patrols, that volunteers used subtle observation and instinct to identify people who might be in need. Highlighting the potential to detect and support individuals who might otherwise go unnoticed due to a lack of visible distress. Used alongside other measures, the Bridge Watch approach may therefore be a valuable strategy for identifying and preventing suicides in public places [3, 32].

### Challenges to service delivery

Despite this potential, challenges around maintaining a comprehensive patrol presence and integrating into support systems are crucial. Effective Bridge Watch coverage was constrained by the volunteer staffing required. In addition, limited awareness of the programme among statutory services highlighted the ongoing challenge of promoting and integrating Bridge Watch within wider service networks.

The Bridge Watch role involves volunteers being confronted with a high variety of complex topics, such as suicidality, mental health crises and substance misuse/intoxication, in public spaces. It is crucial to train and prepare volunteers for the reality of their roles. Our study adds to the importance of context-specific training and the use of real-life examples in training [33]. The use of simulation/role plays has been noted as a promising tool for improvements in attitudes, skills, knowledge and behaviours [34], and our study further supports this.

Our findings highlight the importance of supportive relationships among volunteers in facilitating learning and skill development (see also [35]). However, our findings suggest that the reliance upon more experienced volunteers has the potential for creating a detrimental feedback loop, which may de-skill and impede confidence development. Indeed, a lack of confidence was a barrier to intervention identified in our sample, echoing previous research [21, 33, 36]. While there is evidence that relevant training can result in both short- and long-term increases in knowledge, attitudes to help-seeking, intervention skills and confidence [33, 37], there can be falls in knowledge and self-efficacy from training over time [37]. Our study shows that even with robust training, due to the nature of suicide intervention, concerns over the severity of a mistake (which is a common barrier to intervention by the members of the public [38]), can also be present in trained volunteers, with this resulting in deferral to more experienced others.

### Strengths and limitations

To our knowledge, this study represents the first evaluation of a suicide prevention initiative employing an in-person volunteer presence. Thus, our study adds novel evidence to the nascent fields of bridge-based volunteer suicide prevention interventions and the potential impacts of volunteer-led initiatives in public spaces. Using a qualitative methodology enabled a rich and nuanced understanding of programme implementation and intervention experiences in this context. However, due to the relatively small-scale nature of the Bridge Watch programme, we had a limited sample of both professional practitioners and volunteers to draw on. Due to the way Bridge Watch supports and signposts service users (i.e., that no personal information is collected), it was not possible to speak to people who Bridge Watch had engaged with. The self-selected nature of our sample has clear limitations. We only spoke to volunteers who were actively engaging in Bridge Watch and who consented to take part, resulting in potentially missed perspectives and insights from those who did not engage or dropped out/stopped volunteering. Therefore, we do know how these link to perspectives around support and training that we found. Similar perspectives around service experience were continually present in the participants’ accounts, but that is not to say a larger or more diverse sample would not have produced differing perspectives. Nevertheless, our sample appeared sufficient to provide valuable insights and data to answer our research questions and address our aims.

### Practice recommendations

It is important to target key public sites of potential suicide risk [39], and to ensure that interventions are appropriate, acceptable and effective. Our findings show that Bridge Watch is perceived as having considerable potential in terms of providing suicide intervention and prevention support, as well as numerous wider public health impacts. However, the findings also show several considerations for the development of Bridge Watch. Bridge Watch operates with limited resources and funding, and its ability to develop, expand and provide increased coverage is bound by this. Better integration into local services and support systems may increase the awareness, use and impact of Bridge Watch, but this requires resources, time and capacity for promotion and integration. Further, to achieve greater impact and coverage across the bridges, more volunteer capacity is needed. Expanding Bridge Watch, therefore, requires careful consideration of how the initiative is promoted, ensuring that recruitment efforts are conducted safely and responsibly to avoid unintentionally drawing attention to the bridges as potential suicide locations. Responsible promotion of the initiative can help support sustainable growth while maintaining sensitivity around the sites involved.

Promoting training which reflects actual engagement and interventions in the specific context and conditions of the City of London bridges (e.g., in situ role-plays) is crucial. Additionally, due to the infrequent nature of interventions and concerns over the potential implications of a failed intervention, offering refresher training to reiterate intervention procedures, having regular role plays/simulations to help maintain skills and confidence in the absence of interventions, and assigning roles at the start of patrols may help some volunteers get more experience or confidence. What is important to note is that authenticity, compassion, and being willing to approach and speak to someone has been described as crucial in interventions, more so than having or knowing the right thing to say [21].

Whilst this paper has focused on key qualitative findings, from which we provide some practice recommendations, our wider Bridge Watch evaluation produced various policy and practice recommendations for the Bridge Watch programme (these are detailed in our Supplementary File).

## Conclusion

Bridge Watch appears to be a promising, acceptable, and valued approach to suicide prevention on the City of London bridges, offering benefits both in direct interventions and in its wider public health contributions through preventing harms from other risky behaviours. Its key strengths lie in its proactive, human-centred approach and its capacity to complement statutory services. However, sustaining and scaling the initiative will require development around training design, volunteer recruitment, and stronger integration with support agencies. Further, in the development of such interventions, consideration needs to be given to potential unintended consequences, including the risk of drawing attention to specific locations as sites of suicide.

## Supplementary Information

Supplementary Material 1.

## Data Availability

The datasets used and/or analysed during the current study are available from the corresponding author on reasonable request, and subject to approval from the Sheffield Centre for Health and Related Research (SCHARR) ethics committee at The University of Sheffield.

## Abbreviations

TIDieR: Template for Intervention Description and Replication
CoL: City of London Corporation’s
RNLI: Royal National Lifeboat Institution
PPIE: Patient and Public Involvement and Engagement
SCHARR: Sheffield Centre for Health and Related Research
