# Supplementary material for: Evaluation of a bridge-based suicide intervention programme: findings from a qualitative study with volunteers and professional staff
Source: BMC Public Health. 2026 Mar 11;26:1267. doi: 10.1186/s12889-026-26849-9 (PMC13088732; doi:10.1186/s12889-026-26849-9)
Supplement: Supplementary file 1 — Supplementary Material 1. [file 12889_2026_26849_MOESM1_ESM.docx]

**Supplementary file**

**Title**: Evaluation of a bridge-based suicide intervention programme: findings from a qualitative study with volunteers and professional staff

1. Information sheet (Consent form (professionals/volunteers’ interviews & volunteers diary)
2. Consent form (professionals/volunteers’ interviews & volunteers diary)
3. Topic guides (Professional staff & volunteers)
4. Bridgewatch Volunteer Diary Entry
5. Policy and practice recommendations for the Bridge Watch programme from our wider evaluation
6. Information sheets

**Information Sheet for Bridgewatch Initiative (professional stakeholder interview)**

**Invitation and brief summary**

You are being invited to take part in an evaluation of the Bridgewatch Initiative. Bridgewatch aims to provide a physical presence in the form of teams of volunteers who 'patrol' the areas on and around the bridges of London, and who have a brief to engage or intervene with anybody indicating intent to enter the water, primarily, for reasons of harm (e.g., the purpose of suicide), but also of non-harm (e.g., fun or fall).

We want to speak to people who work within or closely with the Bridgewatch Initiative to hear what they think about it and how it could be made better.

You do not have to take part if you do not want to. To help you decide whether or not to participate, it is important for you to understand why the Bridgewatch evaluation is being done and what it will involve. Please read the following information carefully and take time to decide whether or not you wish to take part. The research team is happy to answer any questions you may have. Contact details of the research team are provided at the end of this information sheet.

**Why are we doing this research?**

The overall aim of the research is to explore the feasibility, acceptability and effectiveness of the Bridgewatch Programme. It will look at the impact of Bridgewatch on the number of people entering the water (for reasons of harm (e.g., suicide) or non-harm (e.g., accidental)) around intervention sites. It will also explore whether Bridgewatch is perceived as an acceptable and beneficial approach from the perspectives of volunteers delivering the programme, key professional stakeholders involved in the initiative, and service users who have engaged with the intervention.

The findings will help shape current and future service delivery and help influence future commissioning intent, as well as having the potential to inform service establishment and delivery in other areas.

**What would taking part involve?**

Taking part would involve having an interview with someone from the research team. This would involve questions about your perceptions and experiences of the Bridgewatch Initiative. For example, we will ask you about:

- Your understanding of the Bridgewatch initiative
- Your experience and perspectives on the recruitment/engagement of volunteers
- If you think the Initiative is meeting its aims and objectives.
- What works well/what could be improved
- Your thoughts on future provision and what you’d like to see changed

The interview will last approximately 60 minutes.

The interviews will be via video call or the telephone - whatever works best for you. The interview will be audio recorded.

We will ask you to complete a consent form before taking part.

**Do I have to take part?**

Taking part in the interview is entirely up to you. You do not have to take part if you do not want to.

It is okay if you change your mind about taking part. You can stop taking part at any point. If you want to stop taking part, any personal details will be deleted.

You can withdraw from the study at any time without giving a reason. This includes after the interview has taken place.

To withdraw from the study, you should contact one of the research team (contact details are at the end of this information sheet). If the interview has taken place, you can contact the research team up to one week after the interview and ask them to delete the recording of the interview. You can do this by providing them with your participation number, which will be written on your consent form. After one week, we will send the recording to be typed up. When we do this, we will remove any personal information, so there will be no way of linking it back to you.

**Are there any possible benefits or downsides to taking part?**

We don’t think there will be any downsides to taking part. We hope that you will enjoy having the time to share your experiences and perspectives on the Bridgewatch Initiative, and opinions about how to make it better.

It is important to know that you do not have to answer any questions you do not want to. If you feel uncomfortable in any way or decide you no longer want to take part, then you can stop at any time without giving a reason. Also, you can skip questions and you do not have to answer a question if you do not want to.

**Is taking part confidential?**

The research team will keep what is said in the interviews confidential, that means we will not tell anyone what is said and any information we use in our research will be anonymised (i.e., your names or anything else that could identify you will not be used). The only time we may have to tell someone what is said is if you say something which suggests there is a risk of significant harm to someone. If this happens we will talk about what will happen next. This follows the Data Protection Act 2018 and the General Data Protection Regulation (GDPR).

Any personal information we collect during the research will be kept strictly confidential and will only be accessible to members of the research team. We’ll have to record some personal details about you when you’re taking part (e.g., names, contact information), but this will be securely stored in password protected spreadsheets on a secure online folder, and these details will be deleted as soon as possible after your interview. Consent forms will be uploaded to a restricted folder on the University of [removed] secure drive. We will write up what we talk about, but nothing will be linked back to you. You will not be named or identified at any point.

All interviews will be audio recorded using a digital recorder. Afterwards, the conversation will be typed up on a computer with all personal information removed so you can’t be identified. All copies of the recording will be destroyed one month after being typed-up. Typed-up recordings will be kept securely in restricted folders on password protected computers that are only accessible by the research team. After the project we will also store the typed-up interviews securely online in a data storage called ORDA; a data repository is a place where research information can be kept after a project has finished for other people to use. This means that other researchers may read the typed-up interviews in the future, but they will not have any way of linking this to you as all personal information will be removed.

All consent forms will be stored securely in restricted folders. Consent forms will be securely stored for 10 years and then deleted.

**Who is responsible for looking after my information?**

The University of [removed] will act as Data Controller for this study. This means they are responsible for looking after your information and using it properly.

**What is the legal basis for processing my personal data?**

Data protection laws say we have to explain to you why we are asking for the personal information we will collect in the interviews. We need this information for research that is a task in the public interest – this means something that is good for the general public.

**What will happen to the findings of this research ?**

The results will be written up in reports and published. We will write up what you tell us so that the Bridgewatch Initiative can be improved, but nothing will be linked back to you.

Your name will not be used in any reports or presentations from this project.

You will be kept informed about the findings if you want to be.

**Has anyone checked this project is okay?**

Before any research is allowed to happen it has to be checked by a group of people called an Ethics Committee. They make sure the research is okay to do. This project has been checked by the University of [removed] Research Ethics Committee through the [removed] Centre for Health and Related Research.

**What if I have questions?**

If you have any questions or are unhappy about anything, please let [removed] or another member of the research team know (contact details are listed at the end of this information sheet). If you would prefer to talk to someone outside of the research team, you can contact [removed]

If you have any issues about the way we handle your personal data, you can contact the University’s Data Protection Officer, [removed]. If you are not satisfied with how your complaint is handled, you may then escalate the complaint to the ICO (Information Commissioner’s Office).

**How can I find out more about this project?**

If you have any questions about the study, you can ask [removed]

If you have any concerns or complaints about the study, you can contact [removed]

**What happens next**

If you are interested in taking part, please contact a member of the research team to arrange a date/time for an interview.

[removed]

Remember, you don't have to take part if you don't want to, and you can change your mind at any time.

Thank you very much for reading this

**Information Sheet for Bridgewatch (Volunteer interview)**

**Invitation and brief summary**

You are being invited to take part in an evaluation of the Bridgewatch Initiative. Bridgewatch aims to provide a physical presence in the form of teams of volunteers who 'patrol' the areas on and around the bridges of London, and who have a brief to engage or intervene with anybody indicating intent to enter the water, primarily, for reasons of harm (e.g., the purpose of suicide), but also of non-harm (e.g., fun or fall).

We want to speak to people who volunteer in the Bridgewatch Initiative to hear what they think about it and how it could be made better.

You do not have to take part if you do not want to. To help you decide whether or not to participate, it is important for you to understand why the Bridgewatch evaluation is being done and what it will involve. Please read the following information carefully and take time to decide whether or not you wish to take part. The research team is happy to answer any questions you may have. Contact details of the research team are provided at the end of this information sheet.

| **Key points**   - You are being invited to take part in an **interview about the Bridgewatch Initiative.** - The interview is about your perspectives and experience of volunteering in the Bridgewatch initiative. - The interview will be **over the phone or via online video call, and will last around 60 minutes**. - The interview will be audio recorded as it is important that we remember what you say to us. - What you say in the interview will be kept confidential. This means we will not tell anyone what you have said. The only time we may have to tell someone what you have said, is if you say something, which suggests a risk of significant harm to you or someone. - Once the interview is completed, the answers will be typed up and safely stored. You will be kept anonymous, this means we won't use your name or anything that could identify you. - **You do not have to take part if you do not want to.** You can also change your mind about taking part at any point. - **You will get a £25 shopping voucher for taking part i**n the interview as a thank you for your time.   **Please read the rest of the information sheet for more details.** |
| --- |

**Why are we doing this research?**

We are doing this research project to understand people's perspectives and experiences of the Bridgewatch initiative. We want to see if the Bridgewatch initiative is working well, and how it can be improved.

You are being asked to take part as you have volunteered with the Bridgewatch Initiative.

**What would taking part involve?**

Taking part would involve having an interview (a chat) with someone from the research team. This would involve questions about your perceptions and experiences of the Bridgewatch Initiative. For example, we will ask you about:

- Your motivations for involvement in the Bridgewatch initiative
- Your experience and perspectives of the training you received
- Your understanding of the Bridgewatch initiative.
- Your perspectives of the effectiveness of the Bridgewatch initiative.
- What works well/what could be improved

The interview will last approximately 60 minutes.

The interviews will be via video call or the telephone - whatever works best for you.

The interview will be audio recorded as it is important that we remember what you say to us.

We will ask you to complete a consent form before taking part. A consent form is a document you sign to say you are happy to take part, that you understand what taking part involves, and that you know what we will do with the information you give us.

You will get a £25 shopping voucher for taking part in the interview as a thank you.

If you are interested in taking part, let the Bridgewatch manager know, and they will pass the research team your contact information. Someone from the research team will be in contact to discuss taking part in more detail, and everything will be organised for you.

**Do I have to take part?**

Taking part in the interview is entirely up to you. You do not have to take part if you do not want to.

It is okay if you change your mind about taking part. You can stop taking part at any point. If you want to stop taking part, any personal details will be deleted.

You can withdraw from the study at any time without giving a reason. This includes after the interview has taken place.

To withdraw from the study, you should contact one of the research team (contact details are at the end of this information sheet). If the interview has taken place, you can contact the research team up to one week after the interview and ask them to delete the recording of the interview. You can do this by providing them with your participation number, which will be written on your consent form. After one week, we will send the recording to be typed up. When we do this, we will remove any personal information, so there will be no way of linking it back to you.

**Are there any possible benefits or downsides to taking part?**

We don’t think there will be any downsides to taking part. We hope that you will enjoy having the time to share your experiences and perspectives on the Bridgewatch Initiative, and opinions about how to make it better.

It is important to know that you do not have to answer any questions you do not want to. If you feel uncomfortable in any way or decide you no longer want to take part, then you can stop at any time without giving a reason. Also, you can skip questions and you do not have to answer a question if you do not want to.

**Is taking part confidential?**

The research team will keep what is said in the interviews confidential, that means we will not tell anyone what is said and any information we use in our research will be anonymised (i.e., your names or anything else that could identify you will not be used). The only time we may have to tell someone what is said is if you say something which suggests there is a risk of significant harm to someone. If this happens we will talk about what will happen next. This follows the Data Protection Act 2018 and the General Data Protection Regulation (GDPR).

Any personal information we collect during the research will be kept strictly confidential and will only be accessible to members of the research team. We’ll have to record some personal details about you when you’re taking part (e.g., names, contact information), but this will be securely stored in password protected spreadsheets on a secure online folder, and these details will be deleted as soon as possible after your interview. Consent forms will be uploaded to a restricted folder on the University of [removed] secure drive. We will write up what we talk about, but nothing will be linked back to you. You will not be named or identified at any point.

All interviews will be audio recorded using a digital recorder. Afterwards, the conversation will be typed up on a computer with all personal information removed so you can’t be identified. All copies of the recording will be destroyed one month after being typed-up. Typed-up recordings will be kept securely in restricted folders on password protected computers that are only accessible by the research team. After the project we will also store the typed-up interviews securely online in a data storage called ORDA; a data repository is a place where research information can be kept after a project has finished for other people to use. This means that other researchers may read the typed-up interviews in the future, but they will not have any way of linking this to you as all personal information will be removed.

All consent forms will be stored securely in restricted folders. Consent forms will be securely stored for 10 years and then deleted.

**Who is responsible for looking after my information?**

The University of [removed] will act as Data Controller for this study. This means they are responsible for looking after your information and using it properly.

**What is the legal basis for processing my personal data?**

Data protection laws say we have to explain to you why we are asking for the personal information we will collect in the interviews. We need this information for research that is a task in the public interest – this means something that is good for the general public.

**What will happen to the findings of this research ?**

The results will be written up in reports and published. We will write up what you tell us so that the Bridgewatch Initiative can be improved, but nothing will be linked back to you.

Your name will not be used in any reports or presentations from this project.

You will be kept informed about the findings if you want to be.

**Has anyone checked this project is okay?**

Before any research is allowed to happen it has to be checked by a group of people called an Ethics Committee. They make sure the research is okay to do. This project has been checked by the University of [removed] Research Ethics Committee through the [removed] Centre for Health and Related Research.

**What if I have questions?**

If you have any questions or are unhappy about anything, please let [removed] or another member of the research team know (contact details are listed at the end of this information sheet). If you would prefer to talk to someone outside of the research team, you can contact [removed]

If you have any issues about the way we handle your personal data, you can contact the University’s Data Protection Officer, [removed]. If you are not satisfied with how your complaint is handled, you may then escalate the complaint to the ICO (Information Commissioner’s Office).

**How can I find out more about this project?**

If you have any questions about the study, you can ask [removed]

If you have any concerns or complaints about the study, you can contact[removed]

**What happens next**

If you are interested in taking part, let the Bridgewatch manager know, and they will pass the research team your contact information.

A member of the research team will be in touch to discuss the research, and to talk about you taking part.

They will speak with you to see if you would like to take part. Interview dates/times will then be arranged for what is suitable for you.

Remember, you don't have to take part if you don't want to, and you can change your mind at any time.

Thank you very much for reading this!

**Information Sheet Bridgewatch (Volunteer Diary/log reflections)**

**Invitation and brief summary**

You are being invited to take part in an evaluation of the Bridgewatch Initiative. Bridgewatch aims to provide a physical presence in the form of teams of volunteers who 'patrol' the areas on and around the bridges of London, and who have a brief to engage or intervene with anybody indicating intent to enter the water, primarily, for reasons of harm (e.g., the purpose of suicide), but also of non-harm (e.g., fun or fall).

We want to explore the thoughts and experiences of Bridgewatch volunteers regarding the patrols they perform. To do this, we’d like Bridgewatch volunteers to complete some reflections and a short list of questions following their patrols. These questions will look at, what things came up in a patrol, how many people were spoken to, what was needed to be done, ect. The aim of this is to identify things that could be changed to help volunteers in their roles.

You do not have to take part if you do not want to. To help you decide whether or not to participate, it is important for you to understand why the Bridgewatch evaluation is being done and what it will involve. Please read the following information carefully and take time to decide whether or not you wish to take part. The research team is happy to answer any questions you may have. Contact details of the research team are provided at the end of this information sheet.

| **Key points**   - You are being invited to take part in **completing a small number of reflections and questions following Bridgewatch patrols.** - You will be asked to **complete and submit four electronic reflection documents** after patrols. They should not take longer than 10 minutes each to complete. - The reflection document contains questions about experiences of patrols, what support was needed to be provided, if you felt able to provide the required support, and how many people you engaged with. - The reflection documents will be sent by you back to the research team. - The information you provide will be kept confidential. This means we will not tell anyone what you have said. The only time we may have to tell someone what you have said, is if you say something, which suggests a risk of significant harm to you or someone. - If you take part you will be kept anonymous, this means we won't use your name or anything that could identify you. - **You do not have to take part if you do not want to.** You can also change your mind about taking part at any point. - **After sending the four reflection documents, you will get a £25 shopping voucher as** a thank you for your time.   **Please read the rest of the information sheet for more details.** |
| --- |

**Why are we doing this research?**

We are doing this research project to understand people's perspectives and experiences of the Bridgewatch initiative. We want to see if the Bridgewatch initiative is working well, and how it can be improved.

You are being asked to take part as you have volunteered with the Bridgewatch Initiative.

**What would taking part involve?**

Taking part would involve completing up to four short reflection documents.

You will be required to complete a small number of reflections and questions following your Bridgewatch patrols. This will be done on a Word document, which you will send back to the research team once completed via email.

Each reflection document should take you no longer than 10 minutes after each patrol.

You will be asked to reflect on the following topics;

- Things that came up during a patrol session, and who was engaged with.
- How did you respond to encountered topics/issues.
- Did you have the skills/knowledge to provide support (what would be good for you/other volunteers to know).
- Your thoughts/feelings after the patrol.

If you decide to take part, a member of the research team will provide you with the reflection document and discuss with you in more detail about how to complete and submit it.

We will ask you to complete a consent form before taking part. A consent form is a document you sign to say you are happy to take part, that you understand what taking part involves, and that you know what we will do with the information you give us.

After sending the four reflection documents, you will get a £25 shopping voucher as a thank you for your time.

If you are interested in taking part, let the Bridgewatch manager know, and they will pass the research team your contact information. Someone from the research team will be in contact to discuss taking part in more detail, and everything will be organised for you.

**Do I have to take part?**

Taking part is entirely up to you. You do not have to take part if you do not want to.

It is okay if you change your mind about taking part. You can stop taking part at any point. If you want to stop taking part, any personal details will be deleted.

To withdraw from the study, you should contact one of the research team (contact details are at the end of this information sheet) and quote provide your participation number, which will be written on your consent form, and any submitted reflection documents will be deleted. This can be done anytime before and upto one week after sending in your last reflection document, as all reflections will be anonymised for analysis at this point. When we do this, we will remove any personal information, so there will be no way of linking it back to you.

**Are there any possible benefits or downsides to taking part?**

We don’t think there will be any downsides to taking part. We hope that you will enjoy having the time to share your experiences and perspectives on the Bridgewatch Initiative and opinions about how to make it better.

It is important to know that your participation is voluntary and if you feel uncomfortable in any way or decide you no longer want to take part, then you can stop at any time without giving a reason. Also, you can skip sections of the reflections and you do not have to answer a question if you do not want to.

**Is taking part confidential?**

The information that you provide will be kept confidential, that means we will not tell anyone what is said and any information we use in our research will be anonymised (i.e., your names or anything else that could identify you will not be used). The only time we may have to tell someone what is said is if you say something which suggests there is a risk of significant harm to someone. If this happens we will talk about what will happen next. This follows the Data Protection Act 2018 and the General Data Protection Regulation (GDPR).

Any personal information we collect during the research will be kept strictly confidential and will only be accessible to members of the research team. We’ll have to record some personal details about you when you’re taking part (e.g., names, contact information), but this will be securely stored in password protected spreadsheets on a secure online folder, and these details will be deleted as soon as possible after your participation. Consent forms will be uploaded to a restricted folder on the University of [removed] secure drive. We will write up what we talk about, but nothing will be linked back to you. You will not be named or identified at any point.

The reflection documents you send to the research team will be encrypted and password protected word documents. The research team will set all this for you, and will inform you of the password. Completed reflection documents will be kept securely in restricted folders on password-protected computers that are only accessible by the research team. After the project we will also store these securely online in a data storage called ORDA; a data repository is a place where research information can be kept after a project has finished for other people to use. This means that other researchers may read the documents in the future, but they will not have any way of linking this to you as all personal information will be removed.

All consent forms will be stored securely in restricted folders. Consent forms will be securely stored for 10 years and then deleted.

**Who is responsible for looking after my information?**

The University of [removed] will act as Data Controller for this study. This means they are responsible for looking after your information and using it properly.

**What is the legal basis for processing my personal data?**

Data protection laws say we have to explain to you why we are asking for the personal information we will collect. We need this information for research that is a task in the public interest – this means something that is good for the general public.

**What will happen to the findings of this research ?**

The results will be written up in reports and published. We will write up what you tell us so that the Bridgewatch Initiative can be improved, but nothing will be linked back to you.

Your name will not be used in any reports or presentations from this project.

You will be kept informed about the findings if you want to be.

**Has anyone checked this project is okay?**

Before any research is allowed to happen it has to be checked by a group of people called an Ethics Committee. They make sure the research is okay to do. This project has been checked by the University of [removed] Research Ethics Committee through the [removed] Centre for Health and Related Research.

**What if I have questions?**

If you have any questions or are unhappy about anything, please let [removed] or another member of the research team know (contact details are listed at the end of this information sheet). If you would prefer to talk to someone outside of the research team, you can contact [removed]

If you have any issues about the way we handle your personal data, you can contact the University’s Data Protection Officer, [removed]. If you are not satisfied with how your complaint is handled, you may then escalate the complaint to the ICO (Information Commissioner’s Office).

**How can I find out more about this project?**

If you have any questions about the study, you can ask [removed]

If you have any concerns or complaints about the study, you can contact [removed]

**What happens next**

If you are interested in taking part, let the Bridgewatch manager know, and they will pass the research team your contact information.

A member of the research team will be in touch to discuss the research, and to talk about you taking part.

They will speak with you to see if you would like to take part, and will provide you with the reflection document and discuss with you in more detail about how to complete and submit it.

Remember, you don't have to take part if you don't want to, and you can change your mind at any time.

Thank you very much for reading this!

2. Consent form

**Bridgewatch Consent Form (Interviews)**

| ***Please tick the appropriate boxes*** | **Yes** | **No** |
| --- | --- | --- |
| **Taking Part in the Project** |  |  |
| I have read and understood the project information sheet dated DD/MM/YYYY and the project has been fully explained to me. (If you will answer No to this question please do not proceed with this consent form until you are fully aware of what your participation in the project will mean.) | ☐ | ☐ |
| I have been given the opportunity to ask questions about the project. | ☐ | ☐ |
| I agree to take part in the project. I understand that taking part in the project involves being interviewed. | ☐ | ☐ |
| I consent to the interview being audio recorded. | ☐ | ☐ |
| I understand that my taking part is voluntary and that I can withdraw from the study at any time; I do not have to give any reasons for why I no longer want to take part and there will be no adverse consequences if I choose to withdraw.  I know I can ask for the recording to be deleted up to seven days after taking part (date: DD/MM/YYYY). | ☐ | ☐ |
| **How my information will be used during and after the project** |  |  |
| I understand my personal details such as name, phone number and email address will not be revealed to people outside the project. | ☐ | ☐ |
| I understand and agree that my words may be quoted in publications, reports, web pages, and other outputs. I understand that I will not be named in these outputs. | ☐ | ☐ |
| I understand and agree that other authorised researchers will have access to this data only if they agree to preserve the confidentiality of the information as requested in this form. | ☐ | ☐ |
| I understand and agree that other authorised researchers may use my data in publications, reports, web pages, and other outputs, only if they agree to preserve the confidentiality of the information as requested in this form. | ☐ | ☐ |
| **So that the information you provide can be used legally by the researchers** |  |  |
| I agree to let The University of [removed] own any outputs that come from this project. | ☐ | ☐ |

________________________ __________________ _____________

Your name Signature Date

________________________ ____________________ _____________

Name of researcher [printed] Signature Date

**Bridgewatch Consent Form (Diary)**

| ***Please tick the appropriate boxes*** | **Yes** | **No** |
| --- | --- | --- |
| **Taking Part in the Project** |  |  |
| I have read and understood the project information sheet and the project has been fully explained to me. (If you will answer No to this question please do not proceed with this consent form until you are fully aware of what your participation in the project will mean.) | ☐ | ☐ |
| I have been given the opportunity to ask questions about the project. | ☐ | ☐ |
| I agree to take part in the project. I understand that taking part in the project involves completing four electronic reflection documents. | ☐ | ☐ |
| I understand that my taking part is voluntary and that I can withdraw from the study at any time; I do not have to give any reasons for why I no longer want to take part and there will be no adverse consequences if I choose to withdraw.  I know I can ask for the reflection documents to be deleted up to seven days after sending in the last reflection document. | ☐ | ☐ |
| **How my information will be used during and after the project** |  |  |
| I understand my personal details such as name, phone number and email address will not be revealed to people outside the project. | ☐ | ☐ |
| I understand and agree that my words may be quoted in publications, reports, web pages, and other outputs. I understand that I will not be named in these outputs. | ☐ | ☐ |
| I understand and agree that other authorised researchers will have access to this data only if they agree to preserve the confidentiality of the information as requested in this form. | ☐ | ☐ |
| I understand and agree that other authorised researchers may use my data in publications, reports, web pages, and other outputs, only if they agree to preserve the confidentiality of the information as requested in this form. | ☐ | ☐ |
| **So that the information you provide can be used legally by the researchers** |  |  |
| I agree to let The University of [removed] own any outputs that come from this project. | ☐ | ☐ |

________________________ ____________________ _____________

Your name Signature Date

________________________ ____________________ _____________

Name of researcher [printed] Signature Date

3. Topic guides

**Topic guide: staff/professional stakeholders**

Note: This topic guide is indicative and will be subject to incremental change from emergent findings/information during the research. This is a guide to the topics to be covered during the interviews, it is not a script, and therefore the order of topics will be flexible.

Introduction:

- Thank you for participating
- Introduction of self
- introduction of study
  - Thank you again for agreeing to take part in this research project.
  - We are speaking to you because you volunteer for the Bridgewatch intervention we are evaluating. We want to explore your thoughts and experiences around what volunteering for the intervention is like, if you think it is being effective, and what’s good or could be improved around the training and support you receive.
- Key points:
  - length of interview (up to 60 minutes)
  - interview as a discussion which will cover key topics
  - no right or wrong answers - exploring perspectives
  - participation is voluntary - right to withdraw participation
  - confidentiality/anonymity
  - how findings will be reported
  - interview will be recorded
  - Questions? Happy to proceed? [Complete and sign consent form if face-to-face interview]
  - There is support available, should you need it following this interview:
    - Samaritans - Call 116 123 - 24/7 Helpline is confidential and free)
    - [offer support information sheet]
  - *start recording*

**Background**

- Where do you work and what is your current job role?
- Can you describe what awareness you have / involvement you have had with the Bridgewatch initiative?
  - *Prompts: establish whether participant is working within the service, or external – if external, how closely do they work?*

**Overview of Bridgewatch**

- Please could you describe what the Bridgewatch initiative is
  - What is it’s intended aims?
  - Why was it set up?
  - How is the service funded?
- From your understanding, how does Bridgewatch run
  - How does a patrol work – what happens during an average patrol
  - Can you describe a typical volunteer interaction with the initiative?
  - How are patrol areas selected
  - What roles do volunteers have
  - Which services do volunteers refer to / engage with?
  - What is the Bridgewatch teams’ relations with external services, how do you work together?
    - Any challenges
    - If external: does Bridgewatch refer to your service, how does this process work and has it had an impact on demand?
- If internal: What is the process for recruiting volunteers, and have you encountered any issues in recruitment and retention of volunteers?
  - How does the training of volunteers work – how has it be set up?
  - How are volunteers supported?
  - Are there any opportunities for volunteers to provide feedback on their experience?

**Effectiveness and Improvement**

- How well is it working
  - What are the intended outcomes
- How far do you feel the service is meeting it’s intended aims?
  - - [preventing entering water]
  - What positive outcomes are being witnessed
  - How do you measure effectiveness
- Is it providing support beyond its original aims
  - Are there other positive outcomes you have witnessed
    - [link to other public health outcomes]
      - General support
      - Presence making people feel safe
      - Directions/advice
- In your opinion, how effective is this intervention in helping people?
  - Do you think it’s working / is it doing what it should be doing
  - *If external service*: how does this service support your work / job role?
  - Can you describe a specific situation where you felt this intervention made a significant difference?

- What aspects of the intervention do you think are most beneficial?
- Has there been any challenges delivering the initiative?
- Are there any areas of the intervention that you think could be improved?
  - Training
  - Delivery
  - Areas of support/scope
  - What are you key areas of learning?
- Has there been any unintended negative outcomes from the initiative?
- If internal: Is the way Bridgewatch runs different to how it was originally planned to run?
  - If so, in what ways

**Close**

- Overall thoughts on the Bridgewatch initiative
- Plans/hopes for the future regarding Bridgewatch
- Any thing we’ve missed you’d like to add/talk about
- Any questions you like to ask me.
  - - [remind about + offer support information sheet]

# **Topic guide: Bridgewatch Volunteers**

Note: This topic guide is indicative and will be subject to incremental change from emergent findings/information during the research. This is a guide to the topics to be covered during the interviews, it is not a script, and therefore the order of topics will be flexible.

Introduction:

- Thank you for participating
- Introduction of self
- introduction of study
  - Thank you again for agreeing to take part in this research project.
  - We are speaking to you because you volunteer for the Bridgewatch intervention we are evaluating. We want to explore your thoughts and experiences around what volunteering for the intervention is like, if you think it is being effective, and what’s good or could be improved around the training and support you receive.
- Key points:
  - length of interview (up to 60 minutes)
  - interview as a discussion which will cover key topics
  - no right or wrong answers - exploring perspectives
  - participation is voluntary - right to withdraw participation
  - confidentiality/anonymity
  - how findings will be reported
  - interview will be recorded
  - Questions? Happy to proceed? [Complete and sign consent form if face-to-face interview]
  - There is support available, should you need it following this interview:
    - Samaritans - Call 116 123 - 24/7 Helpline is confidential and free)
    - [offer support information sheet]
  - *start recording*

**Background**

- Can you tell me about when you first saw or heard about the Bridgewatch service?
- When did you become a volunteer?
- What motivated you to become a volunteer?

- What was the process like becoming a volunteer ,
  - applying to volunteer/getting started/doing the training
  - how long did it take
- How long have you been volunteering?
- Where do you normally do your patrols?
  - How often do you do them
  - How long do they run
  - When
  - Who with (how many people are involved)
- Can you describe your role as a volunteer
  - What are your responsibilities

**Training** (do people feel equipped and prepared)

- Have you received any specific training for this role?
- What was the training like?
  - good quality
  - detailed
  - how long did it take to do
  - how was it delivered
- Does the training cover everything you need to know / does the training reflect the actual experiences of the job role?
  - Are there differences between what you expected / what the training offered, and actually doing the role?
- Do you feel equipped to deliver the role?
  - Is there anything you would change? About the training?
- Do you have any suggestions for additional training or resources that would be beneficial for volunteers using this intervention?
  - Do you have suggestions for how the training could be changed to better reflect your experiences?
- Aside from the training, could the Bridgewatch service provide anything else that would be helpful to support your training and development?

**Bridgewatch experience** (difference between training and real life)

- Can you walk me through a typical volunteering session
  - Fun/boring/busy
  - What do you do in a usual session
    - How many others people are you with
  - Who do you speak to / how many people
  - Do you focus on people at risk of entering the water, or are you providing other support to other people
    - Like what
- [if relevant] Can you walk me through a typical interaction with someone?
  - How do you manage supporting vulnerable/at risk people
    - - During + afterwards
      - How do you manage leaving people after intervening/offering onward support
- Do you feel you have clear steps and guidance around what to do?
  - Have there been any situations you felt unable to deal with?
    - What did you do in this situation?
    - If not, what would you do (e.g., contact someone)
- Do you signpost people to other services after speaking to them?
  - If so what are they?
- Is volunteering what you expected it to be
  - Before you started / from your training

**Effectiveness and improvement**

- In your opinion, how effective is this intervention in helping people
  - Do you think it’s working / is it doing what it should be doing
  - What aspects of the intervention do you think are most helpful?
  - Can you describe a specific situation where you felt this intervention made a significant difference?
- What are some of the most positive outcomes you've seen with this intervention?
  - Are there other positive outcomes
  - [link to other public health outcomes]
    - General support
    - Presence making people feel safe
    - Directions/advice
- What are some of the biggest challenges you've faced while volunteering?
  - Language diversity
  - Sensitive nature of topic
  - Onward support/referral for range of issues
  - Abuse from service users / disengagement
  - Busy = challenge to identify
  - How do you feel about having a fail interaction?
  - The Bridgewatch intervention involves the discussion and support of sensitive topics, do you feel there is adequate support for your own emotional well-being?
    - support before/during/after
- Are there any areas of the intervention that you think could be improved?
  - Do you have the opportunity to raise/suggest these
- What information do you wish you would have known before/when you started
  - What information should other volunteers know
- What impacts, if any, has volunteering had on you?
  - Has volunteering, and doing the training, had an impact on your self-confidence in your own abilities to support people.

**Close**

- What are your future plans for volunteering
- What do you hope to gain from volunteering
- Would you encourage other people to become involved
  - What would you say to them
- Any thing we’ve missed you’d like to add/talk about
- Any questions you like to ask me.
  - - [remind about + offer support information sheet]

4. Bridgewatch Volunteer Diary Entry

Bridgewatch Volunteer Diary Entry

Please complete the below table. Add as much information as you can. You can use bullet points to highlight key points.

Please return this to [*add researcher emai*l].

| **Participant ID (researchers to enter)** |  |
| --- | --- |
| **Diary entry number (1,2,3,4)** |  |
| **Date of patrol** |  |
| **Date of diary entry completion** |  |
| **General reflections of the patrol**  Please provide your reflections of the patrol:  Who did you engage with?  Was it busy/quiet - How many people did you speak to?  What was discussed? How did you feel? |  |
| **What actions were taken as a result of the patrol?**  Did you refer to external services? Provide information or support?  (you can list them if it helps  e.g., 1. What happened, and what you did. 2. What happened, and what you did. |  |
| **Was there anything you felt unable or unequipped to respond to, and how did you do about this?**  Anything new/different encountered?  Anything you need more information on? |  |
| **What worked well/not so well?** |  |
| **Reflecting on your experiences, what would be useful to know for you/other volunteers?** |  |
| **How are you feeling after the session?** |  |
| **Any other thoughts or reflections?** |  |

5. Policy and practice recommendations for the Bridge Watch programme from our wider evaluation

*Enhance volunteer training and support*

Bridge Watch training could be developed through:

- Developing and adding training which reflects actual engagement and interventions in the specific context and conditions of the London bridges (nosiness, busyness from tourists and commuters, the difficulty of identifying people in need, and how these have weather, seasonal, time-of-the-day and day-of-the-week influences).
- Having training focused on the crisis nature of suicide interventions (e.g., more intervention-focused on those actively suicidal, as well as prevention-focused for people ‘reaching out’ for support).
- Using case studies and role plays/simulations based on real-life examples of Bridge Watch interventions so volunteers can learn and reflect on how to respond in different situations.
- Practicing ‘in-situ' roleplays on the bridges to develop knowledge, skills and confidence in intervening in the conditions of the bridges. This may help volunteers better prepare for the realities and practicalities of an intervention.
- Employing mentoring from more experienced volunteers to learn about how they responded in certain situations (including suicide/emergency intervention and non-emergency intervention).
- Offering refresher training to reiterate intervention procedures, or regular role plays/simulations to help maintain skills and confidence in the absence of interventions.
- Working with volunteers to look at frequently encountered challenges during patrols, using this to inform additional training options (e.g., around basic first aid, training around managing ‘non-crisis’ mental health issues, and managing intoxicated individuals).

Support for volunteers could be enhanced by:

- Developing and disseminating more detailed guidance, pathways and processes of volunteer support options, and making this accessible to volunteers (e.g., sent via email, or publicised in the current Bridge Watch volunteer base) would be beneficial to refresh and re-highlight support options.
- Encouraging reflection and debriefing after patrols, especially after 'unresolved' interventions. Encouraging the sharing of reflections may be beneficial for volunteers in learning how to better manage and respond to situations.
- Developing a ‘kit list’ for new volunteers (e.g., waterproof clothes, warm clothes, comfortable shoes, a drink, a notebook and pen, bring your phone, snacks, etc.) to help people be more prepared for the potential realities of the patrols.

*Volunteer confidence and experience*

- The infrequent nature of interventions, concerns over the potential implications of a failed intervention and a tendency for some to defer to more experienced volunteers, limits opportunities for gaining practical experience. Assigning roles at the start of patrols may help some volunteers get more experience or confidence. Encouraging volunteers to approach people of concern, even if it is not a clear suicide intervention, may help build confidence in leading an intervention.
- Re-highlighting misconceptions about suicide discussions (i.e., that there is little harm that can be caused by speaking to someone about suicide intent) may improve confidence.
- Increasing awareness of abilities and limitations around preventing suicide or preventing someone from entering the water (e.g., the limits of what can be done in an intervention), may remove some pressure and expectation, and improve confidence to intervene.

*Improved knowledge of Bridge Watch processes*

- Develop guidance and protocols outlining steps for emergency and non-emergency interventions (i.e., those with no clear and immediate suicide risk), with clearer pathways for less experienced volunteers (e.g., developing guidance with examples based on previous Bridge Watch interactions).
- Disseminate collection of useful phone numbers to all volunteers (e.g., police control room, Royal National Lifeboat Institution).
- Develop knowledge of external support agencies and local support options for onward support/signposting for frequently encountered issues. Knowledge of local support options, or information on how to access local support could be beneficial for volunteers to have access to provide to people spoken to (for example,<https://hubofhope.co.uk/>). This could be a list of resources accessible to volunteers, or a small amount of accessible information (e.g., a prompt card) which could be provided to people after an intervention.
- Highlight policies and processes for recording interventions/patrols, and develop data collection, management and monitoring processes. This may support evidencing wider impacts of Bridge Watch, and targeting of patrols, by accurately recording all interactions and interventions.
- Clarify shift leader roles and responsibilities for volunteers, and empower shift leaders to make decisions and manage situations (to ease workload on the Bridge Watch programme lead ).
- A more formal and structured patrol plan and debrief process in place for each patrol, led by the shift leader, may be beneficial and provide consistency in patrols. This could involve: [patrol planning] check-in, plan and teams, intervention roles, need-to-know information; [patrol debrief] what situations were encountered, what information was needed (e.g., signposting information, etc.), recording support/interventions, processes of support and aftercare.

*Volunteer recruitment and retention*

- Map current volunteer recruitment sources to help establish how well current recruitment strategies are working, and where work can be done to increase recruitment.
- A recruitment and communications strategy could be developed in collaboration between organisations with experience in public messaging around suicide (such as Samaritans), alongside the communications team within the wider Bridge Watch team, to develop a volunteer recruitment strategy (e.g., over social media) which is sensitive and targeted.
- Highlighting a clear training ‘roadmap’ which outlines mandatory and optional training and opportunities for skill development. Presenting the Bridge Watch training package to new/potential volunteers may support recruitment and help show the skills and continuous development they would receive by volunteering.
- Highlighting benefits from volunteering (developing skills in crisis support, improving mental health and wellbeing, building social connections) may support recruitment.
- Highlighting the realities of the volunteering role (e.g., the physicality of patrolling bridges). Providing shadowing opportunities to help people assess if the role is suitable for them.
- Regular updates of outcomes and impacts can help build a collective sense of achievement and contribution, and maintain volunteers' motivation, well-being and awareness of the value of the work.
- Highlighting feedback from other agencies about the role of Bridge Watch in their organisation was also noted as helping highlight the impact and increase volunteer motivation by improving awareness of where Bridge Watch fits into the wider system of support.
- Incentives to support volunteer recruitment/engagement could be considered.

*Collaboration and communication with other services*

- Develop communication and contact with relevant services who operate around the bridges, to improve awareness of the Bridge Watch role. Promotional work with relevant agencies may be beneficial to increased awareness of Bridge Watch, as there suggested little wider knowledge across teams/people with direct engagement or contact with Bridge Watch.
- More formal communication and contact between Bridge Watch and other relevant services may enable better joint working (e.g., sharing patrol times with services beyond the police control room and ‘clocking in’ with other services at the start of patrol) to ensure that other services are aware of when Bridge Watch is operating.
- Despite there being no confirmed risk high frequency locations , there was a suggestion that some bridges are less popular with tourists, so loitering on these can raise attention. Further work around perceived/anecdotal localised and specific ‘risk’ indicators (e.g., transport links, accessibility, views) could be undertaken with volunteers and services operating across the bridges.
- Explore sustainable funding sources with partner agencies to expand programme coverage, provide adequate resources for volunteers, and potentially establish paid support roles for the programme lead. Administration support for the Bridge Watch programme lead may be useful, and allow a separation of tasks around volunteer coordination (arranging the patrol rotas, volunteer recruitment, managing volunteers) and programme coordination (promotion of the programme to key services and stakeholders, fundraising).
